# Impact of a Reminder App on Bladder Filling During Moderately Hypo-Fractionated Radiotherapy for Prostate Cancer: Results of the REFILL-PAC-HYPO Trial

**DOI:** 10.3390/jcm15218559

**Published:** 2026-11-03

**Authors:** Dirk Rades, Stefan Janssen, Laura Doehring, Jan-Dirk Küter, Michael von Staden, Ahmed Al-Salool, Julia Koeck, Justus Domschikowski, Christian Felix Schulz, Jon Cacicedo, Christine Vestergård Madsen, Charlotte Kristiansen

**Affiliations:** 1 Department of Radiation Oncology, University of Luebeck, 23562 Luebeck, Germany; stefan.janssen@uksh.de; 2 Department of Radiation Oncology, University Medical Center Schleswig-Holstein, Luebeck Campus, 23538 Luebeck, Germany; laura.doehring@uksh.de (L.D.); jan-dirk.kueter@uksh.de (J.-D.K.); michael.vonstaden@uksh.de (M.v.S.); ahmed.al-salool@uksh.de (A.A.-S.); 3 Medical Practice for Radiotherapy Rundestrasse, 30161 Hannover, Germany; 4 Department of Radiotherapy, Malteser Hospital St. Franziskus, 24939 Flensburg, Germany; julia.koeck@malteser.org (J.K.); justus.domschikowski@malteser.org (J.D.); 5 Department of Radiation Oncology, University Medical Center Schleswig-Holstein, Kiel Campus, 24105 Kiel, Germany; christian.schulz@uksh.de; 6 Department of Radiation Oncology, Cruces University Hospital, 48903 Barakaldo, Spain; jon.cacicedofernandezbobadilla@osakidetza.eus; 7 Department of Radiation Oncology, Biobizkaia Health Research Institute, 48903 Barakaldo, Spain; 8 Department of Oncology, Vejle Hospital, University Hospital of Southern Denmark, 7100 Vejle, Denmark; christine.vestergaard.madsen@rsyd.dk (C.V.M.); charlotte.kristiansen@rsyd.dk (C.K.)

**Keywords:** bladder volume, hypo-fractionated radiotherapy, propensity score analysis, prostate cancer, reminder app

## Abstract

**Background/Objectives**: Moderate hypo-fractionation is frequently used for irradiation of prostate cancer. Irrespective of dose fractionation, radiotherapy can lead to radiation cystitis. To reduce urinary toxicity, it is important to avoid bladder volumes < 200 mL. This may be facilitated with an app reminding patients to drink water prior to irradiation. This non-randomized multicenter REFILL-PAC-HYPO trial with external control evaluated the association between use of the reminder app and bladder filling during a radiotherapy course. **Methods**: Twenty-two patients receiving hypo-fractionated irradiation (20 × 3.0 Gy) were planned to be supported by the app (the prospective experimental group) and compared to 95 patients who were not supported by the app (the historical control group). The primary endpoint was the number of fractions with a bladder volume < 200 mL. Secondary objectives included satisfaction with the app and its impact on using health technology. **Results**: The median number of fractions with a bladder volume < 200 mL was lower in the app-supported experimental group (13; interquartile range: 6, 18 vs. 19; interquartile range: 14, 20). The Wilcoxon–Mann–Whitney test indicated a significant between-group difference (*p* = 0.0009) with a Hodges–Lehmann estimate of −4 fractions (95% confidence interval: −7 to −2). Supportive analyses used a multivariable negative binomial model (rate ratio: 0.71; *p* = 0.0018) and propensity score analysis (*p* = 0.012). The dissatisfaction rate was 20%; 78.9% of patients stated at least a little impact of trial participation on the use of health technology. **Conclusions**: Given the limitations of this study, the app was associated with fewer fractions with a bladder volume < 200 mL in this cohort. Although patient dissatisfaction was considered acceptable, further optimization of the app would be welcomed. Moreover, the findings of this study require confirmation in a randomized trial that also considers urinary toxicity.

## 1. Introduction

Many patients with localized prostate cancer receive external beam radiotherapy (EBRT), either alone or combined with a brachytherapy boost [1]. For many years, EBRT alone was administered with conventional fractionation (five times 2.0 Gy per week) over seven to eight weeks. The preferred dose-fractionation regime has changed due to three randomized trials that investigated moderately hypo-fractionated radiotherapy with doses per fraction > 2.0 Gy [2,3,4,5,6,7,8]. In 2016, the results of a randomized non-inferiority trial including 3216 patients with localized prostate cancer were published, which compared 74 Gy of conventionally fractionated irradiation over 7.4 weeks to moderate hypo-fractionation with 19 × 3.0 Gy (3.8 weeks) or 20 × 3.0 Gy (4.0 weeks) [2]. The 20 × 3.0 Gy treatment was found to be non-inferior to 74 Gy with respect to clinical or biochemical failure. Toxicities were not significantly different in the three dose-fractionation groups. The authors suggested that 20 × 3.0 Gy should be the new standard approach for localized prostate cancer. In the same year, Lee et al. published their results of a randomized trial non-inferiority including 1115 patients with low-risk tumors [3]. These authors had compared conventional radiotherapy with 73.8 Gy (1.8 Gy per fraction) over 8.2 weeks to moderately hypo-fractionated irradiated with 70 Gy (2.5 Gy per fraction) over 5.6 weeks. The hypo-fractionated regimen turned out to be not inferior to conventional fractionation with respect to disease-free survival at 5 years. However, late genitourinary and gastrointestinal toxicities were more pronounced in patients receiving hypo-fractionated irradiation. In 2024, Lee et al. presented long-term results of their trial [4]. Regarding the estimated disease-free survival at 12 years, the non-inferiority of the hypo-fractionated regimen was confirmed. Grade ≥ 3 late toxicity rates were no longer significantly different. A third randomized non-inferiority trial was performed that included 1,206 patients with intermediate-risk prostate cancer and reported in 2017 [5]. Non-inferiority was shown for 20 × 3.0 Gy over 4 weeks when compared to conventionally fractionated irradiation with 78 Gy (2.0 Gy per fraction) over 8 weeks with respect to biochemical failure at 5 years. Grade ≥ 3 late genitourinary and gastrointestinal toxicities were not significantly different. After the publication of trials, moderately hypo-fractionated radiotherapy has been increasingly used for treatment of localized prostate cancer, in particular for low-risk and intermediate-risk tumors [2,3,4,5,6,7,8,9]. Many centers treat their patients with 20 × 3.0 Gy, which was used in two randomized trials [2,5].

Irrespective of its dose-fractionation concept, EBRT for prostate cancer can lead to significant toxicity, including acute cystitis. Previous studies showed that the risk of radiation-related urinary toxicity, including cystitis, is inversely correlated with the bladder volume, i.e., the filling status of the bladder, at the time of the computed tomography used for treatment planning (planning CT) and during the radiotherapy course [10,11,12]. In the retrospective study of Pisani et al. that investigated 280 prostate cancer patients treated with EBRT, a bladder volume of <200 mL was significantly associated with the occurrence of acute urinary toxicity [10]. In the prospective study of Pinkawa et al. (204 patients), a bladder volume of ≤180 mL was associated with pollakiuria at the end of the EBRT course, and alguria persisted longer [11]. Moreover, Grün et al. found in their prospective study of 193 patients that achieving a reproducible bladder filling above 180 mL and maintaining this volume during the course of radiotherapy via biofeedback resulted in less acute genitourinary toxicity [12]. In two other studies, a bladder volume of >200 mL resulted in less motion of the prostate during radiotherapy sessions, and envisaged dose constraints to reduce urinary toxicity were achieved more often [13,14].

When considering the findings of these studies [10,11,12,13,14], it becomes obvious that it is important to avoid bladder volumes of <200 mL during as many radiation fractions as possible. An idea of how to achieve this goal was the implementation of drinking protocols, instructing patients to drink a pre-defined amount of fluid in the range between 200 mL and 600 mL [12,13,14,15,16,17,18,19,20,21,22,23,24,25,26]. In the corresponding studies, the interval between intake of fluid and the planning CT or radiation fraction ranged between 30 and 60 min. Since following a drinking protocol may have represented a significant challenge for the patients, a reminder app was created. This app had already been successfully tested in healthy volunteers regarding its functionality and practicability [27]. In the REFILL-PAC-HYPO trial, the value of the app was evaluated prospectively [28]. Patients receiving 20 × 3.0 Gy for prostate cancer who were supported by the reminder app were compared to a historical control group treated with the same dose-fractionation regimen but not supported by an app. To achieve the best possible comparability of both groups considering the non-randomized design, an inverse probability of treatment weighting (IPTW) approach was used to match the cohorts.

## 2. Materials and Methods

The REFILL-PAC-HYPO trial is a non-randomized prospective multi-center study, which investigated the impact of a reminder app on the bladder filling status during a course of radiotherapy for localized prostate cancer [28]. Participating centers included three university hospitals (Lübeck, Vejle, and Kiel), one academic teaching hospital (Flensburg), and one private practice (Hannover). These centers contributed eight, seven, one, two and five patients, respectively. Patients in the prospective cohort were recruited between 26 August 2025 and 13 May 2026. They were supported by an app that reminded them 45 min prior to each radiation to drink 300 mL of water. The app was created and maintained by the start-up company Nextlabel OHG from Luebeck, Germany. The rights of the app remain with Nextlabel OHG. After the patients agreed in principle to participate in the REFILL-PAC-HYPO trial, they were informed in detail about the trial and the role and nature of the reminder app. When the bladder volume in the planning CT was found appropriate for the trial, a second conversation with the patients followed, during which written informed consent was obtained. Patients were offered support by medical staff members of the treating department or the Academy of the University Medical Center Schleswig-Holstein regarding download and use of the app. For activation of the app, Nextlabel OHG received the patient’s e-mail address. To ensure protection of this address, a contract, including a data protection concept, was concluded between the University Medical Center Schleswig-Holstein (sponsor of the REFILL-PAC-HYPO trial) and the creator of the app. Nextlabel OHG had neither a role in the development of the trial protocol nor in the handling of study-related data. The patients could set the reminder time separately for each day or for an entire week, depending on the schedule of their radiotherapy course. The patients had to confirm successful intake of water each day, for which they received a puzzle piece as a reward. Time and amount of water intake were assessed by staff members prior to each radiation fraction. The primary objective was the median number of radiation fractions with a bladder volume < 200 mL. Secondary objectives included patient satisfaction with the app and the impact of using the reminder app within the REFILL-PAC-HYPO trial on the use of health technology.

The prospective cohort was compared to a historical control group irradiated for localized prostate cancer since 2022, all but 13 patients in the participating centers in Lübeck (five patients), Vejle (27 patients), Kiel (one patient), and Hannover (49 patients). The other 13 patients were contributed from Barakaldo in Spain. The data of the majority of the patients in the control group were previously analyzed in a retrospective pre-study, amongst other endpoints with respect to the number of radiation fractions with a bladder volume < 200 mL. This was mandatory for the sample-size calculation of the prospective part of the REFILL-PAC-HYPO trial. This pre-study included 76 patients without restriction of the pre-radiotherapy bladder volume. The mean and median numbers of fractions with a bladder volume < 200 mL were 13.7 (standard deviation, SD = 6.7) and 16.0 (interquartile range: Q1, Q3 = 8.0, 19.0), respectively [29]. A pre-radiotherapy bladder volume of <200 mL was a significant prognostic factor in this context. In the corresponding 52 patients with a pre-radiotherapy bladder volume of <200 mL, the mean and median numbers of radiation fractions with a bladder volume < 200 mL were 16.0 (SD = 5.5) and 19.0 (Q1, Q3 = 14.5, 20.0), respectively. These 52 patients from the pre-study were chosen as the control group for the initial version of the protocol of the REFILL-PAC-HYPO trial. When these patients gave written informed consent to radiotherapy, they were also informed about the importance of a full bladder and received standard instructions regarding the intake of fluid (approximately 300 mL).

This trial was performed in accordance with the Declaration of Helsinki, approved by the responsible local ethics committees, and posted on the clinicaltrials.gov website (NCT06784115, posted on 20 January 2025). Preliminary approval with minor comments was obtained from the leading Ethics Committee in Luebeck, Germany, on 17 January 2025 (file 2024-640), and final approval was obtained after successfully addressing the comments on 22 January 2025 (file 2024-640_1). Initial trial-specific criteria for inclusion in the REFILL-PAC-HYPO trial included histologically proven prostate cancer, indication for definitive hypo-fractionated irradiation, possession of and ability to use a smartphone, and a bladder volume of <200 mL according to the planning CT. Patients receiving irradiation of pelvic lymph nodes and patients with expected non-compliance were not eligible. Moreover, to qualify for the analyses, patients had to have been treated with 20 × 3.0 Gy and received a cone-beam computed tomography (CBCT) scan prior to each radiation fraction. Contouring of the bladder and measurement of the bladder volume was performed by a selected number of experienced and instructed staff members. Blinding and central quality reviews were not performed.

The initial control group consisted of 52 patients who received hypo-fractionated irradiation between 2022 and 2024 and met the inclusion criteria stated above [29]. Twenty-three of the 52 patients were contributed from Hannover, 20 patients from Vejle, eight patients from Barakaldo, and one patient from Lübeck. During the performance of the REFILL-PAC-HYPO trial, it was realized that a considerable number of patients had a pre-radiotherapy bladder volume greater than 200 mL but less than 250 mL. Therefore, it was decided to adjust the corresponding inclusion criterion, i.e., to use <250 mL instead of <200 mL. This adjustment was based on a retrospective study, which included the 52 above-mentioned plus 39 additional patients treated between 2022 and 2025 [30]. The additional 39 patients were contributed from Hannover (26 patients), Vejle (seven patients), Barakaldo (five patients), and Kiel (one patient). They received the same standard information and instructions regarding the intake of fluid as the initial 52 patients. Median number of fractions with a bladder volume < 200 mL was 19.0 in both patients with a bladder volume of <250 mL prior to radiotherapy (range: 14.0–20.0) and a bladder volume of <200 mL prior to radiotherapy (range: 15.0–20.0). The mean numbers of radiation fractions with a bladder volume < 200 mL were almost identical, i.e., 15.9 (SD = 5.6) vs. 16.0 (SD = 5.5).

**Figure 1:**
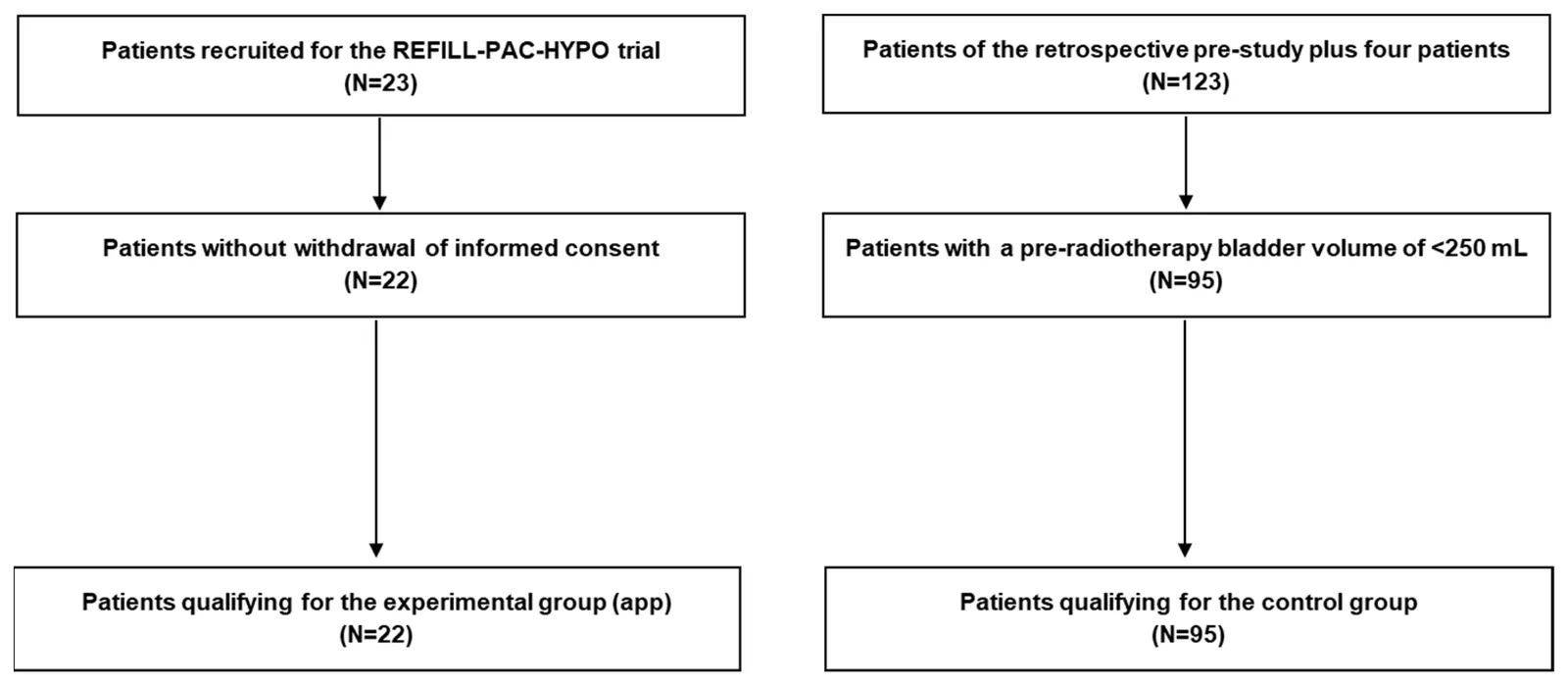
Flow diagram showing patients who qualified for the experimental and the control group.

The adjustment was approved by the responsible Ethics Committee on 26 September 2025 (file 2024-640_2). Although the study was finally approved by the responsible Ethics Committee in January 2025, recruitment started only at the end of August 2025, one month prior to the amendment coming into force. During this month, two patients were not included because their initial bladder volume was between 200 mL and <250 mL. Of the 22 patients included in the analyses, five patients were enrolled prior to the commencement of the amendment. Ninety-one patients of the second pre-study qualified for the control group (Figure 1) [30]. Following approval of the amendment, the sample-size calculation was repeated using the expanded historical control group of 91 patients to ensure 90% power to reach statistical significance with a two-sided Wilcoxon–Mann–Whitney U test and a 5% significance level. The revised calculation required 21 evaluable patients and resulted in a recruitment target of 23. Four patients from Luebeck who met the inclusion criteria for the prospective trial but were not able or did not agree to participate in the REFILL-PAC-HYPO trial were added to the control group, which finally included 95 patients. Two out of 22 patients (9.1%) in the experimental group and twelve out of 95 patients (12.6%) in the control group with an initial bladder volume between 200 mL and <250 mL were included.

Out of the 23 patients enrolled, 22 patients qualified for Full Analysis Set (intention-to-treat analysis) and the analyses with respect to the study endpoints (Figure 1). One patient withdrew his informed consent prior to the start of radiotherapy. Moreover, in one case, the app could only be used for 16 radiation fractions (fractions 5 to 20); in another case, it did not work for any of the 20 fractions. Seventeen out of the 20 patients with a properly working app had no problems with drinking 300 mL water 45 min prior to radiotherapy. One patient had to urinate prior to irradiation on six out of 20 days. Two patients drank the required amount of water 20 min instead of 45 min prior to irradiation on one and two days, respectively. All patients received the total number of 20 CBCT scans required for determination of the bladder volume according to the study protocol [28]. The baseline characteristics of these patients are summarized in Table 1.

### Statistical Considerations and Analyses

All statistical analyses were performed in the Full Analysis Set, which included the 22 patients with available patient-level data and an evaluable endpoint. In accordance with the Full Analysis Set described in the study protocol, these patients were retained in the analysis because data for the primary endpoint were available [28]. Accordingly, no exclusion was made solely on the basis of having fewer than 20 fractions. Missing values for the body-mass index in the historical control group (Table 1) were handled by creating an explicit missing category within the corresponding categorical variable, thereby avoiding exclusion of patients from the analysis solely because information regarding the body-mass index was unavailable. The baseline characteristics were summarized descriptively according to the treatment group. Categorical variables were reported as counts and percentages. Count variables were summarized using the median number of fractions plus Q1, Q3, minimum and maximum, mean number of fractions plus standard deviation, and standard error. Unadjusted between-group differences were summarized as mean differences with Satterthwaite 95% confidence intervals. Stratified descriptive analyses were performed across prespecified baseline covariate strata. These analyses were considered exploratory and descriptive.

The primary endpoint was the number of radiotherapy fractions with a bladder volume below 200 mL. The protocol-specified primary unadjusted comparison between groups was based on the Wilcoxon–Mann–Whitney test, and the Hodges–Lehmann estimator was used as the primary nonparametric estimate of the between-group difference. The Hodges–Lehmann estimate is based on pairwise between-group differences rather than the difference between group medians. Descriptive endpoint distributions were summarized separated by treatment group using nonparametric statistics, particularly median, interquartile range, and range values. Mean differences with Satterthwaite confidence intervals were retained for a sensitivity analysis. Subgroup analyses comparing patients supported by the app with patients in the control group were performed for the investigated baseline characteristics shown in Table 1. Because the endpoint was defined at the patient level as the number of fractions with a bladder volume < 200 mL during a 20-fraction observation window, a protocol-specified multivariable count model was used as a supportive analysis of the conditional mean count. Baseline factors showing a tendency toward between-group heterogeneity were identified using categorical tests, with a threshold of *p* < 0.20 for inclusion. These factors, together with treatment group, were entered into a Poisson regression model with a log link. Over-dispersion was assessed using the Pearson chi-square statistic divided by the residual degrees of freedom. In the case of over-dispersion, the Poisson model was replaced by a negative binomial model. Effects were reported as rate ratios with 95% confidence intervals. Because the negative-binomial distribution does not explicitly impose the upper bound of 20, this model was regarded as a supportive approximation rather than as a model of the full bounded outcome distribution. Primary inference remained based on the prespecified nonparametric patient-level comparison. Propensity score analysis was performed as a supportive covariate-adjusted sensitivity analysis because the study compared a prospective cohort with a historical control group. The propensity score was estimated using Firth-penalized logistic regression, with treatment group as the dependent variable. The prespecified baseline covariates were initial bladder-volume category, age category, Karnofsky performance status, body-mass-index category, pre-radiotherapy prostate-volume category, pre-radiotherapy prostate-specific antigen category, Gleason score, primary tumor stage, prostate cancer risk group, and antihormonal treatment. Body-mass index was modeled using the categories < 30 kg/m^2^, ≥30 kg/m^2^, and missing.

Firth-penalized logistic regression was selected to improve stability in the setting of a small prospective cohort and categorical baseline covariates.

Stabilized inverse probability weights targeting the average treatment effect were calculated from the marginal treatment-group probabilities and individual propensity scores, without trimming or truncation. Propensity score overlap, weight distributions, effective sample sizes, and covariate balance were assessed. Balance was evaluated using absolute standardized mean differences, with values < 0.10 indicating adequate descriptive balance; for multilevel characteristics, the maximum absolute value across levels was used. The IPTW effect was estimated as the difference in stabilized weighted means between the experimental and historical control groups using a robust variance estimator [32].

Moreover, satisfaction of the patients with the reminder app was assessed at the day of the last radiation fraction. Assessment was performed with a questionnaire based on a previously described and established approach [33,34] that included 12 contrasting pairs (attributes related to the app). For each of these pairs, the patient could give 1 to 7 points, depending on agreement or disagreement with the attribute. Positive attributes included comprehensible, easy to learn, valuable, exciting, interesting, predictable, supportive, simple, safe, activating, as expected, and clear. Negative attributes were incomprehensible, difficult to learn, substandard, boring, uninteresting, unpredictable, impeding, complicated, unsafe, soporific, not as expected, and confusing. The positive attributes comprehensible, easy to learn, supportive, simple, safe, and clear were considered more important than the other “softer” attributes. Scores were adapted, so that more points represented a greater level of satisfaction for each attribute. The maximum possible score was 12 × 7 = 84 points. In accordance with other prospective trials and after discussions with clinical experts in the field, a patient was rated as satisfied if they achieved a mean score of at least 4.0 points (a total score of ≥48 points, if all 12 attributes were rated by the patient) [28,35,36]. In case of a dissatisfaction rate of >20%, the current version of the app needed some adjustments but was considered useful in principle. If the dissatisfaction rate was greater than 40%, the app was not considered useful at all and could not be recommended for further investigation. Adverse events were assessed weekly during the course of radiotherapy.

## 3. Results

For the primary endpoint, the distribution of fractions with a bladder volume < 200 mL was shifted toward fewer inadequate volume fractions in the experimental group (reminder app). The median number of fractions with a bladder volume of <200 mL was 13.0 (Q1, Q3 = 6.0, 18.0; range: 1−20) in the experimental group and 19.0 (Q1, Q3 = 14.0, 20.0; range: 0−20) in the control group. Without the two patients for whom the app did not work properly, the median and mean numbers of fractions with a volume < 200 mL in the experimental group were 12.5 (Q1, Q3 = 6.0, 17.0; range: 1−20) and 11.3 (±6.2), respectively. In the protocol-specified nonparametric comparison, the Wilcoxon–Mann–Whitney test yielded a *p*-value of 0.0009, and the Hodges–Lehmann estimate for the experimental group minus the control was −4 fractions (95% CI: −7 to −2). The corresponding mean values were 11.7 (SD = 6.4) and 16.0 (SD = 5.5), respectively. The mean-difference analysis was treated as a sensitivity analysis and was consistent with the primary nonparametric result (mean difference: −4.25; 95% confidence interval: −7.26 to −1.25; *p* = 0.0072). Adverse events related to the app were not observed.

### 3.1. Initial Unadjusted Analysis

**Figure 2:**
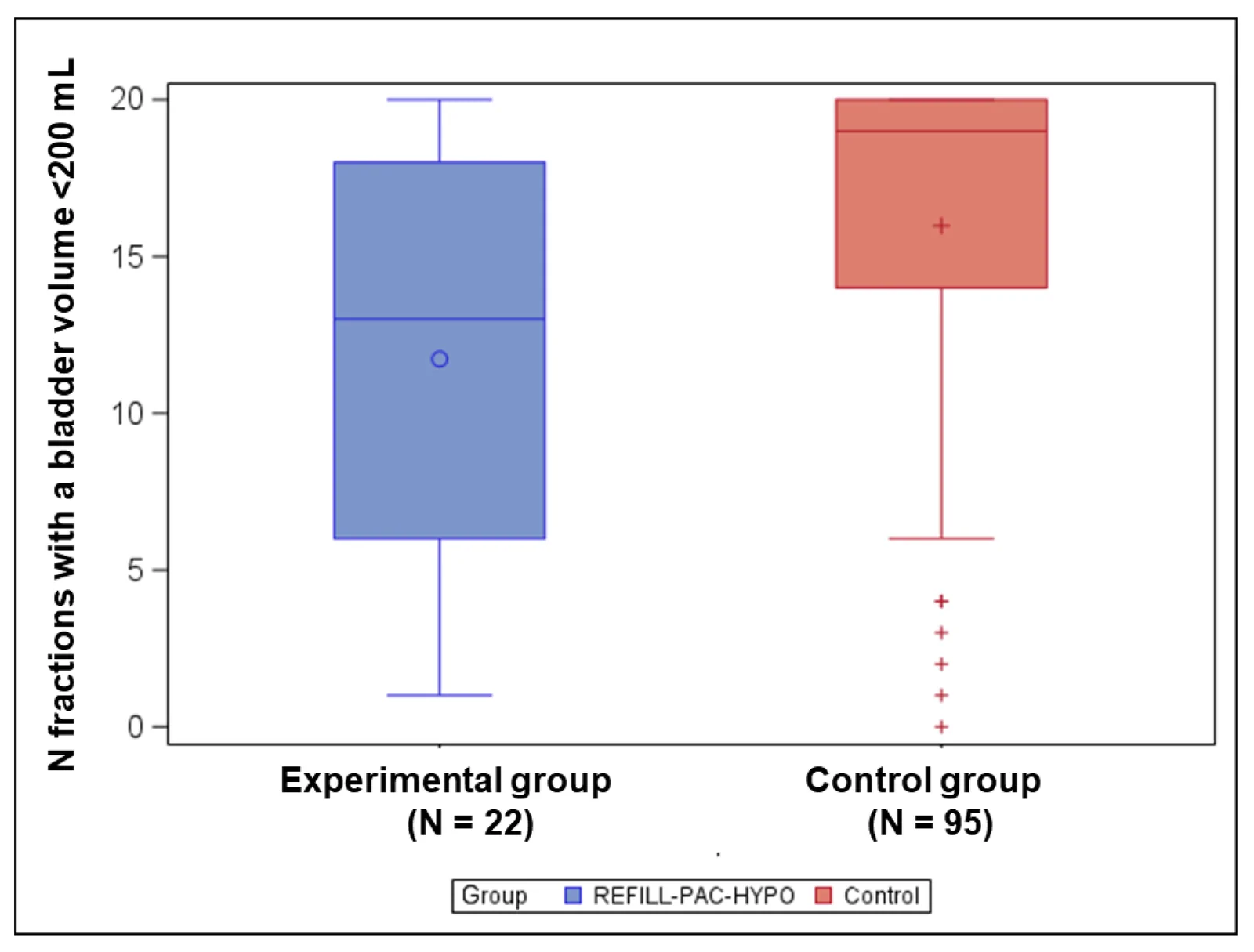
Box-and-whisker plot comparing the number of fractions with a bladder volume < 200 mL between the experimental group supported by the app and the control group without the app.

The box-and-whisker plot in Figure 2 visualizes the distribution of the number of radiotherapy fractions with a bladder volume < 200 mL in the experimental and control groups. The Hodges–Lehmann difference describing the location shift between the experimental and the control group was −4 with a 95% confidence interval of −7 to −2. When applying the Wilcoxon–Mann–Whitney test, a (two-sided) *p*-value of 0.0009 was obtained.

**Figure 3:**
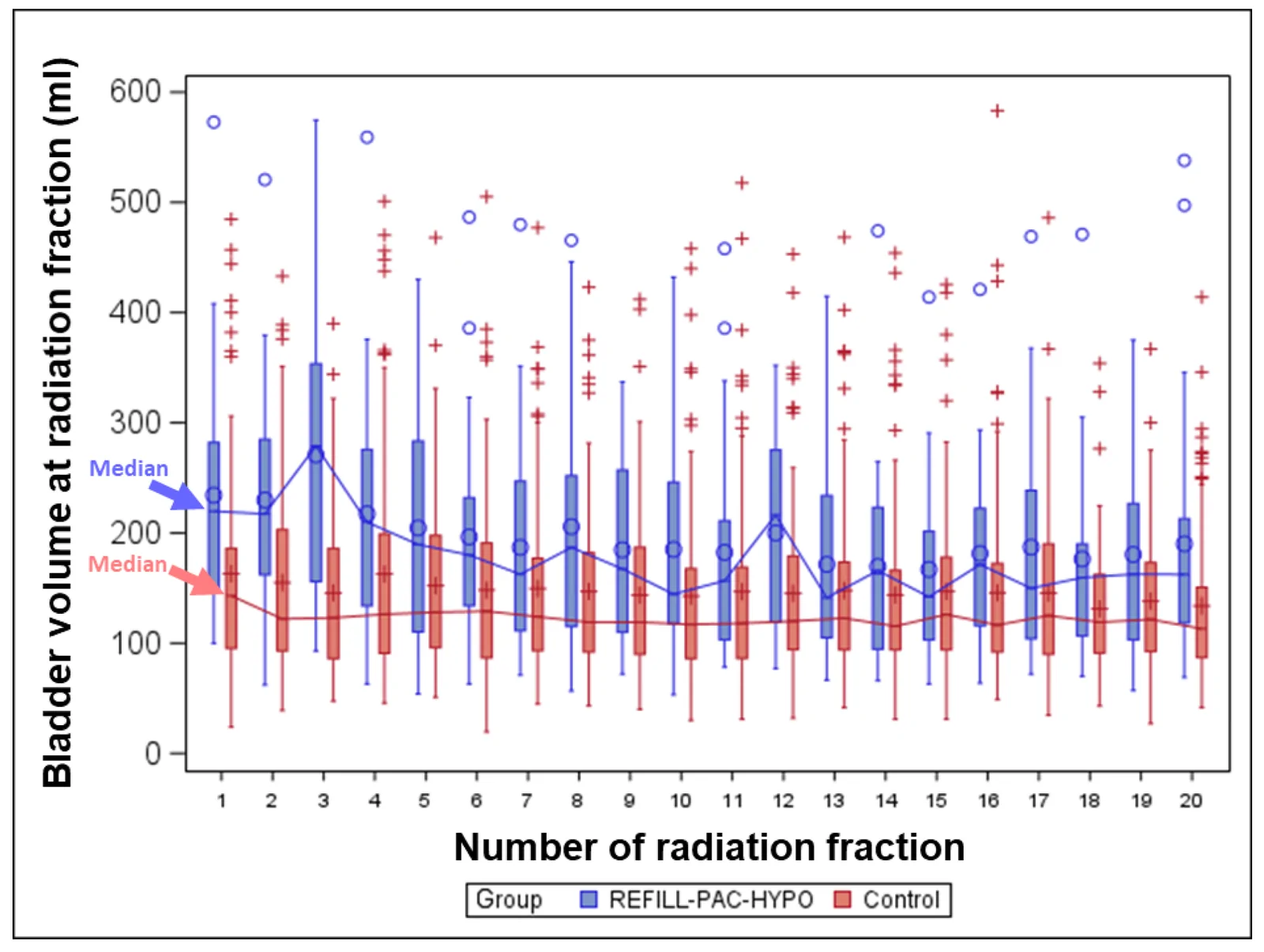
Box-and-whisker plot of bladder volumes at each fraction for both groups, including mean values plus SDs and median values plus ranges (thin vertical lines).

In addition, descriptive analyses were performed for the course of bladder volumes during the series of 20 fractions (Figure 3). The mean bladder volumes (plus SDs) of each fraction of the experimental and the control group are summarized in Table 2.

**Figure 4:**
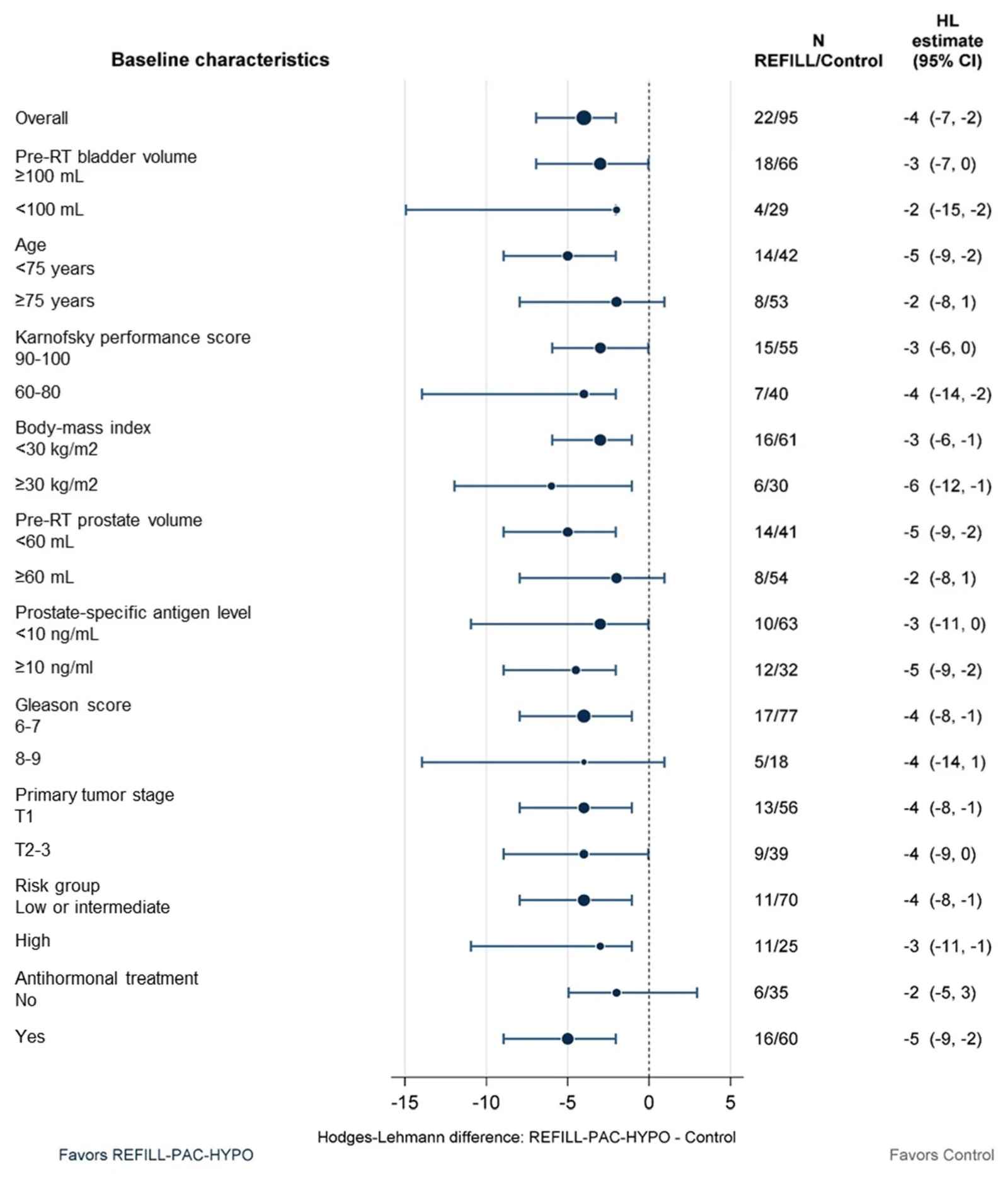
Forest plot of Hodges–Lehmann estimates for the number of fractions with a bladder volume < 200 mL. Estimates are shown for the experimental group minus controls; horizontal bars indicate 95% confidence intervals (negative values favor the experimental group).

**Figure 5:**
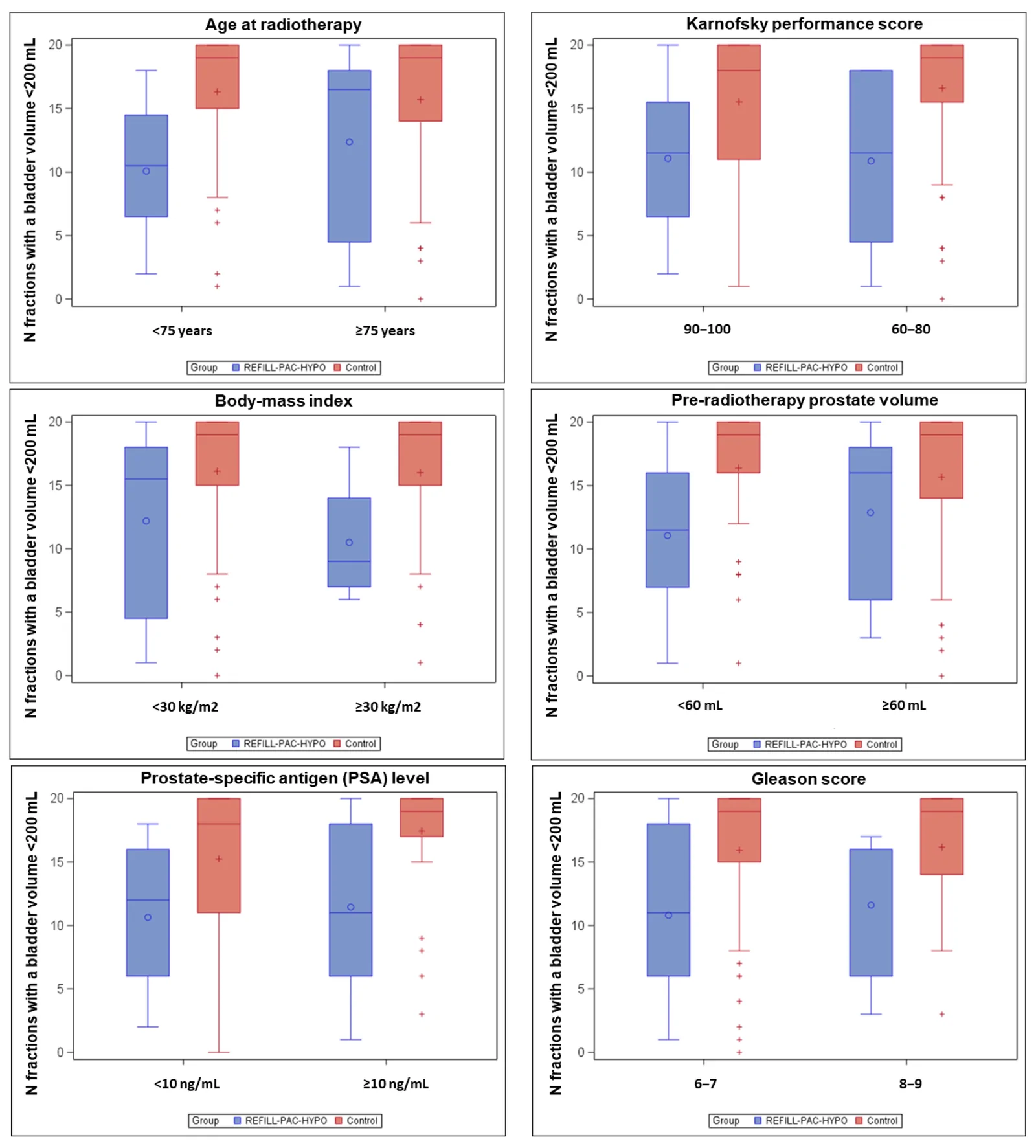

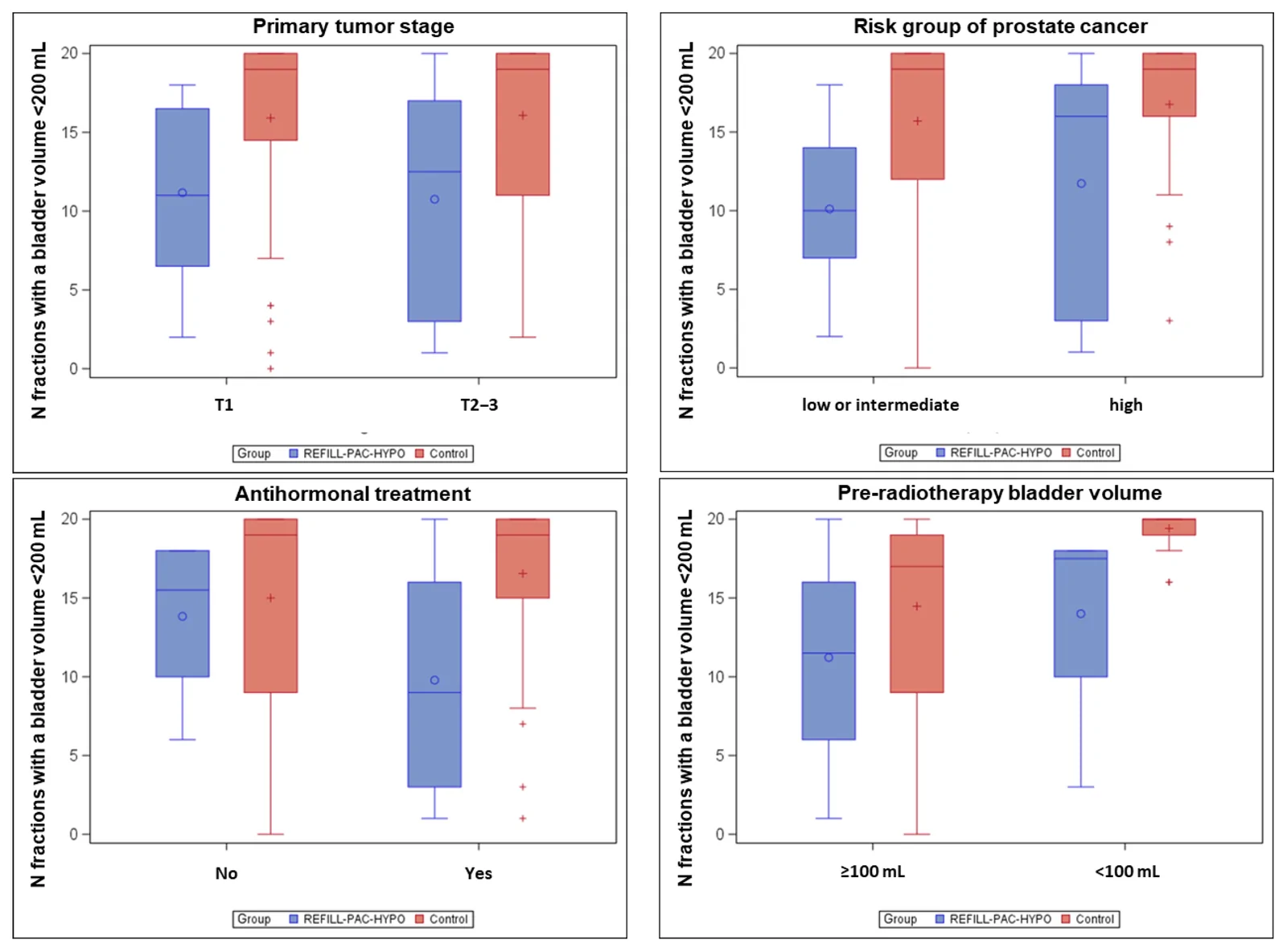
Box-and-whisker plots of the number of fractions with a bladder volume < 200 mL in the experimental (with app) and the control (without app) group, stratified by baseline characteristics.

Exploratory subgroup analyses were used to evaluate the consistency of the primary nonparametric treatment effect across baseline characteristics. Associated Hodges–Lehmann estimates are shown in Figure 4, whereas Table 3 focuses on group-wise nonparametric descriptive endpoint distributions. Box-and-whisker plots visualized underlying subgroup-specific distributions (Figure 5).

As a sensitivity analysis, mean differences with Satterthwaite confidence intervals and *t*-tests were evaluated to assess whether the primary findings were robust under a mean-based summary (Table 4).

### 3.2. Multivariable Modeling

A protocol-specified multivariable count model was fitted as a supportive analysis. Per protocol, variables with a tendency toward heterogeneity (*p* < 0.20) were entered into a multivariable count model together with the binary treatment factor. This approach was used to keep the number of parameters in the model low, considering the small sample size of 22 patients in the experimental group. Center and calendar period were not included, nor was the pre-radiotherapy bladder volume, which was significantly associated with the number of fractions with a bladder volume < 200 mL in our pre-study [29]. A Poisson model was fitted first. However, the Pearson chi-square divided by the residual degrees of freedom was approximately 2.22, which indicated relevant over-dispersion. Therefore, the protocol-defined negative binomial model was used for a supportive analysis of the conditional mean count. The model included treatment group, age category, prostate-volume category, risk group, and prostate-specific antigen level (Table 5).

In the negative binomial model, the estimated rate ratio for the experimental group vs. the historical control group was 0.71, with a 95% confidence interval of 0.57 to 0.88 and a *p*-value of 0.0018. This indicates that, after adjustment for age category, prostate-volume category, risk group of prostate cancer, and PSA level, the expected number of fractions with a bladder volume < 200 mL was approximately 29% lower in the experimental group supported by the reminder app when compared to the control group without the app. The adjusted estimate was directionally consistent with the primary nonparametric analysis.

### 3.3. Propensity Score Analysis

Propensity scores ranged from 0.038 to 0.740 in the historical control group and from 0.123 to 0.603 in the experimental group. Stabilized weights ranged from 0.844 to 3.126 and from 0.312 to 1.528, respectively. The corresponding effective sample sizes were 86.1 patients in the historical control group and 18.8 patients in the experimental group. Weighting improved the balance for most of the measured baseline characteristics. However, residual imbalance remained for age, body-mass index, pre-radiotherapy prostate-specific antigen level, prostate volume, and prostate cancer risk group.

The stabilized weighted mean number of fractions with a bladder volume < 200 mL was 12.29 in the experimental group and 16.06 in the historical control group. The IPTW-adjusted mean difference was −3.77 fractions (robust 95% CI: −6.70 to −0.84; *p* = 0.0116). The weighted estimate was consistent in direction with the primary nonparametric analysis, although complete balance of the measured baseline characteristics was not achieved.

### 3.4. Secondary Objectives and Acute Urinary Toxicity

Twenty out of the 22 patients (90.9%) completed the questionnaire evaluating patient satisfaction with the app (secondary objective of the study). Each of the twelve attributes were addressed by all 20 patients. Since the dissatisfaction rate (average score < 4.0 points) was 20.0% (four patients), the reminder app did not need further optimization according to the pre-defined acceptable rate of dissatisfaction. No patient achieved an average satisfaction score of maximum possible 7.0 points. Average scores were between 6.0 and 6.9 points in five patients (25.0%), between 5.0 and 5.9 points in seven patients (35.0%), between 4.0 and 4.9 points in four patients (20.0%), between 3.0 and 3.9 points in three patients (15.0%), and between 2.0 and 2.9 points in one patient (5.0%). In addition, the mean scores (plus SDs) per attribute were calculated (Table 6). The only attribute with a mean score < 4.0 points was exciting (negative = boring). When considering the six attributes that may be regarded more important with respect to the quality of the app, namely, comprehensible, easy to learn, supportive, simple, safe, and clear, the mean scores ranged between 4.85 and 6.50 and were ≥5.50 points for five attributes.

Another secondary objective was the potential impact of the participation in the REFILL-PAC-HYPO trial and the use of the reminder app on the general use of health technology. To obtain this information, the patients were asked whether they felt more confident (safer) using their smartphone due to the participation in the REFILL-PAC-HYPO trial (question 1). Moreover, they were asked whether, due to the participation in the trial, they used their smartphone more often in general (question 2), more often to learn about prostate cancer (question 3), or more often to learn about other health aspects such as nutrition or physical activity (question 4). For each question, three answers (no, a little more often, or much more often) were possible.

Nineteen patients (86.4%) completed this questionnaire. Question 1 was answered with “no” by ten patients (52.6%) and with “a little more often” by nine patients (47.4%). Answers for question 2 were “no” in 12 cases (63.2%), “a little more often” in six cases (31.6%) and “much more often” in one case (5.3%). Question 3 was answered with “no” by seven patients (36.8%), with “a little more often” by eight patients (42.1%), and with “much more often” by four patients (21.1%). Answers for question 4 were “no” in nine cases (47.4%), “a little more often” in six cases (31.6%) and “much more often” in four cases (21.1%). When combining “a little more often” and “much more often”, participation in the trial, including being supported by the reminder app, had at least a little impact on the use of health technology in 47.4% (question 1), 36.8% (question 2), 63.2% (question 3), and 52.6% (question 4) of the patients.

In patients in the experimental group, acute urinary toxicity was assessed prospectively and documented in the case report form. Grading was performed using version 5.0 of the Common Terminology Criteria for Adverse Events [37]. Urinary toxicity was observed in 16 out of the 22 patients (72.7%). Fourteen patients reported pollakiuria and/or nocturia, which was mild (grade 1) in seven patients and moderate (grade 2) in seven patients. Dysuria/pain was reported by four patients (grade 1 in three patients, grade 2 in one patient). One patient developed urinary retention requiring intervention (grade 3). In total, maximum acute toxicity was grade 1 in nine out of 22 patients (40.9%), grade 2 in six patients (27.3%), and grade 3 in one patient (4.5%).

## 4. Discussion

Prostate cancer is the most common cancer type in men in Western countries [38]. Many patients with localized disease are treated with EBRT alone. With respect to the dose per fraction, treatment regimens are classified as conventionally fractionated (dose per fraction = 1.8 to 2.0 Gy), moderately hypo-fractionated (dose per fraction = 2.4 to 3.4 or 2.2 to 4.0 Gy), and ultra-hypo-fractionated (dose per fraction ≥5.0 Gy or >4.0 Gy) irradiation [39,40,41]. Since three randomized trials demonstrated moderately hypo-fractionated irradiation given over approximately 4 weeks to be not inferior compared to conventional fractionation given over 7 to 8 weeks, moderate hypo-fractionation is used for the majority of patients with localized prostate cancer assigned to radiotherapy, commonly with 20 × 3.0 Gy over 4.0 weeks [2,3,4,5,6,7,8].

However, each type of EBRT for prostate cancer bears the risk of urinary toxicity, including acute cystitis, which can be quite stressful for the affected patients. Three previous studies found that urinary toxicity is increased in case of inappropriate filling of the bladder prior to and during the radiotherapy course [10,11,12]. Cut-off values of the bladder volume used in these studies were 180 mL or 200 mL. Two other studies recommended bladder volumes > 200 mL, which were associated with less motion of the prostate and better achievement of the dose constraints defined to reduce the risk of urinary toxicity [13,14]. Thus, avoidance or at least a reduction in the number of fractions with bladder volumes of <200 mL during the course of radiotherapy is very important. To achieve this goal, drinking protocols for the patients have been introduced, which sometimes may be difficult to follow. In the study of Smith et al., patients followed the bladder filling guidelines at 91 out of 192 fractions (47.4%) [14]. Moreover, a drinking protocol may not have a significant impact on the bladder filling status. For example, in the study of Braide et al., estimated median bladder volumes were 120 mL (95% confidence interval: 93 to 154 mL) in patients who received instructions to drink 300 mL of fluid 60 min prior to irradiation compared to 123 mL (95% confidence interval: 98 to 155 mL) in patients who were allowed to maintain a comfortable bladder filling [15]. Doherty et al. compared the results of two studies [19]. In the first study, patients were asked to drink 450 mL and were allowed to have comfortable bladder filling. In the second study, patients received written information regarding bladder filling and were asked to drink 350 mL. Bladder filling consistency was better in the second study. However, the authors stated in their conclusions that patients were not able to maintain constant bladder filling during the course of radiotherapy [19]. In order to possibly improve the bladder filling status, the potential value of a reminder app was investigated in the present study [27,28]. Twenty-two patients were eligible for the Full Analysis Set (intention-to-treat analysis) of the prospective REFILL-PAC-HYPO trial and received the app. However, in one case the app could not be used for the initial four radiation sessions, and in another case it could not be used prior to any fraction. Patients in the Full Analysis Set (N = 22) were compared to retrospective historical control group of 95 patients, who also received moderately fractionated irradiation but were not supported by a reminder app. The primary endpoint was the median number of fractions with a bladder volume < 200 mL. In the primary nonparametric analysis, the distribution of this endpoint was shifted toward fewer bladder-volume fractions < 200 mL in the experimental group. The Hodges–Lehmann estimated indicated an absolute reduction of four fractions. The negative binomial model provided a supportive covariate-adjusted analysis of the patient-level count and yielded an estimate directionally consistent with the primary nonparametric comparison. However, the model does not explicitly impose the upper bound of 20, and its result should therefore be interpreted as an approximation of the conditional mean count rather than as a complete representation of the endpoint distribution.

The supportive IPTW analysis yielded an adjusted estimate that was consistent in direction with the primary nonparametric result. Weighting improved the balance for most measured baseline characteristics, but residual imbalance remained for several covariates.

The difference was more pronounced when the two patients who faced problems regarding the usability of the app were not considered. Unfortunately, the findings of the REFILL-PAC-HYPO trial cannot be reasonably compared to the results of previous studies that investigated the value of drinking protocols, due to significantly different study designs and objectives. These patients experienced 20 and 12 fractions, respectively, with a bladder volume < 200 mL. Another noteworthy result is the fact that the app appeared to have a more pronounced impact on bladder filling in patients aged < 75 years when compared to older patients. In both age groups, the median number of fractions was 19.0 in the control without support by the app. In the experimental group who used the app, the median values in elderly and younger patients were different, i.e., 16.5 vs. 11.5 fractions. In the group of younger patients, the decrease in the median number of fractions with a bladder volume < 200 mL was even statistically significant (*p* = 0.0036). These findings may suggest that a separate app for elderly patients may be useful.

Secondary objectives included satisfaction with the app and the impact of participating in the prospective trial and receiving the app on the patient’s behavior regarding the use of health technology. The dissatisfaction rate was 20%, i.e., exactly at the pre-defined limit where it was considered acceptable, and can, therefore, be considered sufficiently low. Remarkably, when looking at the results for single attributes, the worst average scores per attribute were found for “exciting” vs. “boring” (3.95 points) and “interesting” vs. “uninteresting” (4.30 points), i.e., for attributes not directly related to the usability or practicability of the app. Moreover, when considering the six attributes that may be regarded more important with respect to the quality of the app, namely, comprehensible, easy to learn, supportive, simple, safe, and clear, the mean scores ranged between 4.85 and 6.50 and were ≥5.50 points for five attributes. Despite the fact that the result regarding the pre-specified definition of satisfaction indicated that modification of the app is not necessary, adjustments based on the patients’ ratings should be performed to obtain an optimal tool in the future. Moreover, when interpreting the satisfaction rates stated in this trial, one should be aware of the fact that the cut-off value of satisfaction represents a study-specific threshold rather than a validated measure of satisfaction. Regarding the impact on the patient’s attitude toward the use of health technology, at least some impact was indicated by 36.8% to 63.2% of the patients. More than half of the patients stated an increased use of their smartphone to gather information about prostate cancer or other aspects of health. However, since these rates were obtained using an unvalidated, uncontrolled questionnaire, they need to be interpreted with caution and should be considered simply as participants’ perceptions. Overall, the reminder app appeared to show some promising results with respect to the primary and secondary objectives. However, due to the limitations of this study stated in the next paragraph, additional studies and further optimization of the app are required. Moreover, the reminder app may be supplemented by other patient-facing digital tools explored in urology [42].

### Limitations

A significant limitation is due to the study design. Instead of a randomized trial with proper stratification of the baseline characteristics, the study compared a prospective cohort (REFILL-PAC-HYPO, the experimental group) to a retrospective group of patients (the control group). Although baseline heterogeneity was assessed and both multivariable and propensity score analyses were performed, residual imbalance remained for some covariates and unmeasured differences between the prospective and historical cohorts cannot be excluded. Moreover, eligibility for the experimental group required possessing a smartphone and being able to use it properly. Since this criterion was not necessary for inclusion in the control group, there may have been selection bias. The fact that some patients in both groups had an initial bladder volume between 200 mL and <250 mL may also have led to bias. The fact that patients in the experimental group were treated more recently than the patients in the control group may have created bias as well. Moreover, patients in the prospective part of this study (the experimental group) may have changed their habits due to participation in a clinical trial (the Hawthorne effect). Another possible source of selection bias might have been introduced by the fact the one out of the six involved centers contributed patients only to the experimental group and another center only to the control group. Additional limitations include the facts that the staff members who performed the contouring of the bladder and the measurement of bladder volume were not blinded to the group or study period (observer bias), that central quality reviews were not performed, and that center and calendar period were not considered in the analyses. Furthermore, there was an imbalance between the size of the treatment groups, which consisted of 22 and 95 patients, respectively, limiting the precision of subgroup-specific estimates. Therefore, subgroup analyses should be primarily interpreted as exploratory and as an assessment of consistency. These limitations support the need for prospective randomized validation of the results. Such a trial must evaluate the impact of the use of a reminder app on direct clinical outcomes such as radiation-related urinary toxicity. Since all patients in both treatment groups were treated with moderately hypo-fractionated EBRT alone, the results may not be valid for other radiotherapy approaches, such as conventionally fractionated EBRT alone over 7 to 8 weeks, EBRT combined with a brachytherapy boost, brachytherapy alone, or stereotactic body radiation therapy [39,43,44,45,46,47]. Moreover, because urinary toxicity was assessed only in the prospective experimental group, the data of this study could not demonstrate an effect of the reminder app on toxicity outcomes.

## 5. Conclusions

In this non-randomized prospective evaluation with a historical control group, use of a reminder app was associated with significantly better filling of the bladder during the radiotherapy course. The supportive analyses yielded estimates in the same direction as the primary nonparametric analysis but could not fully exclude residual or unmeasured confounding. When considering the pre-specified cut-off value for an acceptable dissatisfaction rate, the app did not require further optimization at this stage. Despite these promising results regarding the value of the reminder app during radiotherapy of prostate cancer, the results of this study must be viewed with caution. Since the study has some limitations, its results should be considered hypothesis-generating, and the qualities of the app need to be confirmed in a randomized trial. Since the bladder filling status must be considered a surrogate parameter, the impact of the app on urinary toxicity must be addressed in a future trial.

## Figures and Tables

**Table 1:** Distribution and comparison of baseline characteristics (*p*-values obtained from the chi-square test) and between-group heterogeneity (*p* < 0.20).

Characteristic	Experimental Group *n* Patients (%)	Control Group *n* Patients (%)	*p*-Value	Heterogeneity
Age <75 years ≥75 years	14 (63.6) 8 (36.4)	42 (44.2) 53 (55.8)	0.100	Yes
Karnofsky performance score 90–100 60–80	15 (68.2) 7 (31.8)	55 (57.9) 40 (42.1)	0.375	No
Body-mass index <30 kg/m^2^ ≥30 kg/m^2^ Missing data	16 (72.7) 6 (27.3) 0 (0.0)	61 (64.2) 30 (31.6) 4 (4.2)	0.607	No
Pre-RT prostate volume <60 mL ≥60 mL	14 (63.6) 8 (36.4)	41 (43.2) 54 (56.8)	0.083	Yes
Prostate-specific antigen level <10 ng/mL ≥10 ng/mL	10 (45.5) 12 (54.5)	63 (66.3) 32 (33.7)	0.069	Yes
Gleason score 6–7 8–9	17 (77.3) 5 (22.7)	77 (81.1) 18 (18.9)	0.767 *	No
Primary tumor stage [31] T1 T2–3	13 (59.1) 9 (40.9)	56 (58.9) 39 (41.1)	0.990	No
Risk group Low or intermediate High	11 (50.0) 11 (50.0)	70 (73.7) 25 (26.3)	0.030	Yes
Antihormonal treatment No Yes	6 (27.3) 16 (72.7)	35 (36.8) 60 (63.2)	0.397	No
Pre-RT bladder volume ≥100 mL <100 mL	18 (81.8) 4 (18.2)	66 (69.5) 29 (30.5)	0.246	No

RT: Radiotherapy; * Fisher’s exact test.

**Table 2:** Descriptive analyses of the mean bladder volumes plus standard deviations (SDs), stratified by radiation fraction, for the experimental and the control group.

Number of Fraction	Experimental Group Mean Value (SD)	Control Group Mean Value (SD)
1	234.3 (115.5)	163.1 (97.0)
2	229.9 (108.5)	154.9 (90.4)
3	270.9 (124.3)	145.6 (78.2)
4	217.4 (113.3)	162.8 (102.3)
5	204.6 (109.1)	152.2 (78.1)
6	196.4 (100.9)	148.2 (85.1)
7	186.9 (105.9)	149.6 (82.2)
8	205.7 (123.1)	146.9 (80.1)
9	184.7 (83.7)	143.6 (79.3)
10	185.1 (94.4)	142.7 (85.5)
11	182.4 (102.2)	146.7 (92.3)
12	200.0 (86.6)	145.2 (82.0)
13	171.5 (99.1)	147.8 (85.0)
14	169.6 (93.9)	143.7 (85.0)
15	166.9 (82.9)	147.0 (80.7)
16	181.3 (84.0)	145.8 (90.5)
17	187.1 (107.2)	145.5 (80.5)
18	176.4 (91.4)	131.1 (56.4)
19	180.5 (95.1)	138.0 (65.1)
20	190.1 (125.2)	133.8 (72.9)

**Table 3:** Nonparametric descriptive distribution of the primary endpoint in the experimental group and the control group in the entire cohort and stratified by baseline characteristics.

Characteristic	Experimental Group Median (Q1, Q3)	Control Group Median (Q1, Q3)
Entire cohort	13.0 (6.0, 18.0)	19.0 (14.0, 20.0)
Age <75 years ≥75 years	11.5 (7.0, 16.0) 16.5 (4.5, 18.0)	19.0 (15.0, 20.0) 19.0 (14.0, 20.0)
Karnofsky performance score 90–100 60–80	14.0 (7.0, 18.0) 7.0 (3.0, 18.0)	18.0 (11.0, 20.0) 19.0 (15.5, 20.0)
Body-mass index <30 kg/m^2^ ≥30 kg/m^2^ Missing data	15.5 (4.5, 18.0) 9.0 (7.0, 14.0) No missing data	19.0 (15.0, 20.0) 19.0 (15.0, 20.0) 14.5 (7.5, 20.0)
Pre-RT prostate volume <60 mL ≥60 mL	11.5 (7.0, 16.0) 16.0 (6.0, 18.0)	19.0 (16.0, 20.0) 19.0 (14.0, 20.0)
Prostate-specific antigen level <10 ng/mL ≥10 ng/mL	11.0 (6.0, 17.0) 13.0 (8.0, 18.0)	18.0 (11.0, 20.0) 19.0 (17.0, 20.0)
Gleason score 6–7 8–9	12.0 (7.0, 18.0) 16.0 (6.0, 16.0)	19.0 (15.0, 20.0) 19.0 (14.0, 20.0)
Primary tumor stage [31] T1 T2–3	12.0 (7.0, 17.0) 14.0 (3.0, 18.0)	19.0 (14.5, 20.0) 19.0 (11.0, 20.0)
Risk group Low or intermediate High	12.0 (7.0, 18.0) 16.0 (3.0, 18.0)	19.0 (12.0, 20.0) 19.0 (16.0, 20.0)
Antihormonal treatment No Yes	15.5 (10.0, 18.0) 11.5 (4.5, 17.5)	19.0 (9.0, 20.0) 19.0 (15.0, 20.0)
Pre-RT bladder volume ≥100 mL <100 mL	11.5 (6.0, 16.0) 17.5 (10.0, 18.0)	17.0 (9.0, 19.0) 20.0 (19.0, 20.0)

RT: Radiotherapy.

**Table 4:** Sensitivity analyses of mean numbers of fractions with a bladder volume of <200 mL plus standard deviations (SDs) in the entire cohort and stratified by baseline characteristics.

Characteristic	Experimental Group Mean Value (SD)	Control Group Mean Value (SD)	Mean Difference (95% Confidence Interval)
Entire cohort	11.7 (6.4)	16.0 (5.5)	−4.3 (−7.3 to −1.25)
Age <75 years ≥75 years	11.4 (5.8) 12.4 (7.7)	16.3 (5.2) 15.7 (5.7)	−5.0 (−8.6 to −1.4) −3.3 (−9.8 to +3.2)
Karnofsky performance score 90–100 60–80	12.6 (5.9) 9.9 (7.3)	15.5 (5.3) 16.6 (5.7)	−2.9 (−6.5 to +0.6) −6.7 (−13.5 to −0.0)
Body-mass index <30 kg/m^2^ ≥30 kg/m^2^ Missing data	12.2 (6.9) 10.5 (4.8) No missing data	16.1 (5.2) 16.0 (5.8) 13.8 (7.3)	−3.9 (−7.8 to −0.0) −5.5 (−10.6 to −0.4)
Pre-RT prostate volume <60 mL ≥60 mL	11.1 (6.3) 12.9 (6.8)	16.4 (5.0) 15.7 (5.8)	−5.3 (−9.2 to −1.5) −2.8 (−8.6 to +3.0)
Prostate-specific antigen level <10 ng/mL ≥10 ng/mL	11.1 (6.7) 12.3 (6.3)	15.2 (5.8) 17.4 (4.5)	−4.1 (−9.1 to +0.8) −5.2 (−9.4 to −1.0)
Gleason score 6–7 8–9	11.8 (6.5) 11.6 (6.6)	15.9 (5.6) 16.2 (5.2)	−4.2 (−7.7 to −0.6) −4.6 (−12.6 to +3.4)
Primary tumor stage [31] T1 T2–3	11.8 (5.9) 11.6 (7.4)	15.9 (5.6) 16.1 (5.4)	−4.1 (−7.8 to −0.3) −4.5 (−10.3 to +1.3)
Risk group Low or intermediate High	11.7 (5.8) 11.7 (7.2)	15.7 (5.8) 16.8 (4.5)	−4.0 (−8.0 to +0.1) −5.0 (−10.1 to −0.0)
Antihormonal treatment No Yes	13.8 (4.8) 10.9 (6.8)	15.0 (6.3) 16.6 (4.9)	−1.2 (−6.3 to +4.0) −5.6 (−9.4 to −1.8)
Pre-RT bladder volume ≥100 mL <100 mL	11.2 (6.2) 14.0 (7.3)	14.5 (5.9) 19.4 (1.1)	−3.2 (−6.6 to +0.1) −5.4 (−17.1 to +6.3)

RT: Radiotherapy.

**Table 5:** Results of the multivariable analysis using a negative binomial model, including treatment group and heterogeneous baseline characteristics.

Term	Rate Ratio	95% Confidence Interval	*p*-Value *
Experimental vs. control group	0.71	0.57–0.88	0.0018
Age group (≥75 vs. <75 years)	0.97	0.81–1.16	0.722
Prostate volume (≥60 vs. <60 mL)	1.00	0.85–1.17	0.973
Risk group (high vs. low or intermediate)	1.02	0.83–1.26	0.827
Prostate-specific antigen level (≥10 vs. <10 ng/mL)	1.13	0.95–1.34	0.167

* Likelihood ratio tests.

**Table 6:** Patient satisfaction with the reminder app.

Positive Attribute	Mean Score	Standard Deviation
Comprehensible	6.50	0.74
Easy to learn	5.95	1.56
Supportive	5.55	1.50
Simple	6.45	1.16
Safe	4.85	2.37
Clear	5.50	1.96
Valuable	4.25	1.73
Exciting	3.95	1.60
Interesting	4.30	1.62
Predictable	5.55	1.88
Activating	4.55	1.56
As expected	5.10	2.12

## Data Availability

Further information regarding the protocol of the REFILL-PAC-HYPO trial can be found at the clinicaltrials.gov website (NCT06784115, posted on 20 January 2025). The study results are included in this article and will not be shared.

## Abbreviations

CBCT	Cone-beam computed tomography
CT	Computed tomography
EBRT	External beam radiotherapy
IPTW	Inverse probability of treatment weighting
OHG	Offene Handelsgesellschaft
RT	Radiotherapy
